# The role of the tumor microenvironment in HNSCC resistance and targeted therapy

**DOI:** 10.3389/fimmu.2025.1554835

**Published:** 2025-04-01

**Authors:** Zhaomeng Guo, Kang Li, Xiaotong Ren, Xijia Wang, Dunhui Yang, Shibo Ma, Xianhai Zeng, Peng Zhang

**Affiliations:** Department of Otolaryngology, Longgang Otolaryngology hospital & Shenzhen Key Laboratory of Otolaryngology, Shenzhen Institute of Otolaryngology, Shenzhen, Guangdong, China

**Keywords:** tumor microenvironment, HNSCC, target therapy, resistance, TAMs

## Abstract

The prognosis for head and neck squamous cell carcinoma (HNSCC) remains unfavorable, primarily due to significant therapeutic resistance and the absence effective interventions. A major obstacle in cancer treatment is the persistent resistance of cancer cells to a variety of therapeutic modalities. The tumor microenvironment (TME) which includes encompasses all non-malignant components and their metabolites within the tumor tissue, plays a crucial role in this context. The distinct characteristics of the HNSCC TME facilitate tumor growth, invasion, metastasis, and resistance to treatment. This review provides a comprehensive overview of the HNSCC TME components, with a particular focus on tumor-associated macrophages (TAMs), regulatory T cells (Tregs), myeloid-derived suppressor cells (MDSCs), cancer-associated fibroblasts (CAFs), the extracellular matrix, reprogrammed metabolic processes, and metabolic products. It elucidates their contributions to modulating resistance to chemotherapy, radiotherapy, targeted therapy, and immunotherapy in HNSCC, and explores novel therapeutic strategies targeting the TME for HNSCC management.

## 1 Introduction

Currently, about 90% of head and neck cancers are classified as head and neck squamous cell carcinoma (HNSCC) (1). HNSCC is the sixth most prevalent cancer globally, with over 870,000 new cases diagnosed and more than 450,000 deaths annually (2). This malignancy primarily affects the mucosal surfaces of four key anatomical regions: the oral cavity, sinuses, pharynx, and larynx. The principal risk factors contributing to the development of HNSCC include tobacco use, excessive alcohol consumption, and human papillomavirus (HPV) infection (3). HNSCC is frequently diagnosed at a locally advanced or distant metastatic stage, which significantly compromises patients’ quality of life. The disease is characterized by a high propensity for metastasis and recurrence, a poor response to conventional therapies, and notable resistance (4, 5). The five-year survival rate post-diagnosis is approximately 50%, with nearly 30% of patients experiencing treatment failure and cancer recurrence (6, 7). Consequently, there is an urgent need to develop novel and therapeutic strategies for HNSCC or enhance the sensitivity of existing treatments to overcome resistance.

**Figure 1:**
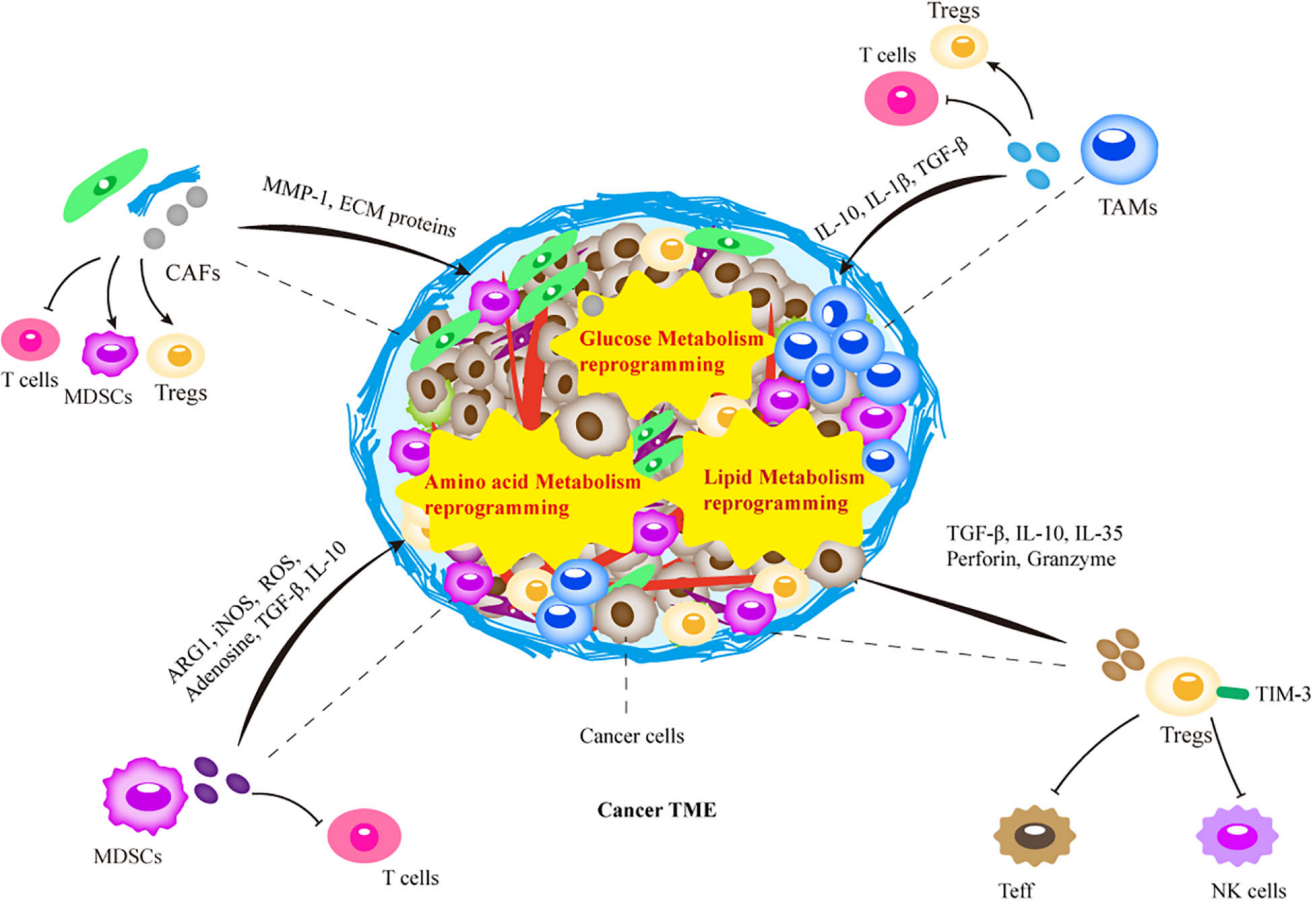
Schematic representation of the tumor microenvironment (TME). The TME is a highly complex ecosystem composed of non-cancerous cells and extracellular components surrounding the tumor. The intricate interactions between these TME elements are involved in the resistance to tumor therapy, which determines the efficacy of tumor therapy. Treated tumors form unique treatment-resistant TMEs through recruitment of immunosuppressive immune cells, or metabolic reprogramming. The recruited immune cells further promote the growth of suppressive immune cells through the release of various cytokines, chemokines, and other cells interactions, together with metabolic reprogramming in TME. Ultimately, this leads to tumor therapy resistance through factors such as physical barriers and immunosuppression. MDSC, myeloid-derived suppressor cells; Treg, regulatory T lymphocytes; TAM, tumor-associated macrophages; CAF, cancer-associated fibroblasts; NK, natural killer cells; Teff, T effector cells.

Previous research has predominantly concentrated on tumor cells. Nevertheless, emerging studies suggest that tumorigenesis, metastasis, and drug resistance in HNSCC may be attributed to interactions between the surrounding stromal tissue and the cells comprising the tumor microenvironment (TME) (8, 9). The TME is a highly complex ecosystem consisting of non-cancerous cells and extracellular components surrounding the tumor (10). Specifically, non-cancerous cells include immune cells such as tumor-associated macrophages (TAMs), myeloid-derived suppressor cells (MDSCs), regulatory T cells (Tregs), dendritic cells (DCs) and natural killer (NK) cells, and non-immune cells such as cancer-associated fibroblasts (CAFs) and endothelial cells (ECs) (11). Non-cellular components of the TME contain collagen, laminin, fibronectin, elastin and various physical/chemical parameters. These diverse cellular subsets and components infiltrate the tumor, interacting with tumor cells and with each other through complex networks (**Figure 1**). HNSCC may evade immune system recognition and elimination, activate immune suppression, and facilitate tumor progression. In summary, the TME provides a conducive environment for tumor progression, metastasis, and the development of drug resistance (12).

A comprehensive understanding of the biological characteristics of HNSCC, particularly the interactions between cancer cells and their surrounding TME, is important for the development of innovative therapeutic approaches, preventing treatment failure, and enhancing treatment sensitivity in patients. Notably, recent therapeutic strategies targeting components of the TME have demonstrated increased efficacy and improved sensitivity to tumor treatment. This review discusses the role of TME components in conferring resistance to tumor treatment.

## 2 Impact of cells and extracellular matrix in the TME on tumor drug resistance

The TME not only facilitates tumor development but also undergoes adaptive changes induced by tumors to support their progression. Therefore, elucidating the molecular mechanisms involved in this progression is crucial for the discovery of more effective cancer therapies.

### 2.1 TAMs

Macrophages, as pivotal elements of the immune system, are responsible for recognizing, phagocytizing, and eliminating bacteria and foreign substances (13). During tumorigenesis, macrophages differentiate into TAMs, which constitute significant components of the innate immune system within the TME (14). TAMs exhibit high plasticity and are influenced by stimuli from tumor cells, leading to their classification into pro-inflammatory M1 and immune-tolerant M2 phenotypes (15). Emerging evidence suggests that secretions or exosomes from tumor cells can alter the transcriptional programs of TAMs, shifting them from M1 to M2 phenotypes (16). A high infiltration of TAMs is frequently correlated with poor clinical outcomes across various cancers and is thought to diminish the efficacy of standard therapies, such as radiotherapy, chemotherapy, and targeted therapy. Following treatment, TAMs accumulate within the TME, contributing to tumor recurrence. M2-type TAMs are particularly associated with promoting tumorigenesis and drug resistance, inducing Th2 responses, and producing anti-inflammatory cytokines (17).

In HNSCC, TAMs play a significant role in treatment resistance. Docetaxel, a mitotic inhibitor, is utilized clinically for various cancers, including HNSCC (18). The incorporation of docetaxel into concurrent chemoradiotherapy and induction chemotherapy represents an innovative treatment approach; however, resistance remains a significant challenge (19). Hsieh CY et al. found that IL-1β secreted by macrophages enhances HNSCC resistance to docetaxel through the SOD2/CAT-ICAM1 signaling pathway. IL-1β is a multifunctional cytokine involved in hematopoiesis, inflammatory responses, immune activities, and drug resistance. Researchers have demonstrated that IL-1β activates both the NF-κB and MAPK pathways, leading to the upregulation of adhesion molecules such as ICAM1 and VCAM1. This activation amplifies and sustains the response to IL-1β, thereby enhancing tumor resistance to docetaxel (20). Gefitinib, a widely used molecularly targeted chemotherapy agent that inhibits the epidermal growth factor receptor (EGFR), has been shown to be influenced by TAMs (21). TAMs secrete various cytokines, including TGF-β and IL-6, which facilitate tumor-promoting activities and immune suppression within the TME (22). Additionally, TAMs secrete prostaglandins and COX-2, both of which play significant roles in immunosuppression (23). TGF-β is capable of modulating the function of programmed cell death protein 1 (PD-1) and PD-1 ligand (PD-L1) by regulating PD-L1 expression. Furthermore, TGF-β not only suppresses T cell activity but also recruits Tregs and MDSCs, thereby further promoting immune suppression (24). Studies have also indicated that prostaglandin E2 induces the expression of Foxp3, which is critical for the differentiation of Tregs (25). Moreover, TAMs have been observed to express PD-L1 in various tumor types. In HPV-related HNSCC, TAMs activated by tumor-derived IL-10 mediate CD8+ T cell dysfunction through PD-1/PD-L1 interactions. TAMs further support cancer cells by producing immunosuppressive cytokines such as IL-10, as well as anti-inflammatory cytokines and chemokines that inhibit T cell activity, thereby contributing to immune suppression (26). Some studies suggest a positive feedback loop between TAMs and Tregs, which amplifies the immunosuppressive effects of TAMs and enhances resistance to immunotherapy. TAMs recruit Tregs to the TME via chemokines such as CCL5, CCL20, and CCL22. Tregs exert immunosuppressive effects by inhibiting effector T cells and dendritic cells, thereby promoting anti-apoptotic effects and increasing cancer cell survival (27). In laryngeal squamous cell carcinoma, Tregs can directly promote the differentiation of monocytes into immunosuppressive TAMs, further contributing to immune suppression (28).

### 2.2 MDSCs

MDSCs originate from hematopoietic stem cells in the bone marrow and are classified into two main types: monocytic MDSCs and granulocytic/polymorphonuclear MDSCs (PMN-MDSCs) (29). These cells can accumulate in various locations in response to pro-inflammatory molecules produced by tumor cells or host cells within tumor tissues and organs (30). MDSCs are primarily characterized by their immunosuppressive capabilities. They predominantly target T cells, thereby playing a critical role in facilitating tumor progression. MDSCs achieve immune suppression by inhibiting T cell activation and promoting Tregs. Within the TME, MDSCs are recruited, developed, and differentiated, ultimately executing functions that are intricately linked to tumor development, metastasis, and resistance to therapeutic interventions (31). While MDSCs are present in low numbers in healthy individuals, their numbers are significantly increased in cancer patients (32). The abundance of MDSCs in circulation and at tumor sites is inversely correlated with the efficacy of anti-tumor therapies and is associated with poor prognosis. Consequently, MDSCs are considered as valuable prognostic biomarkers in cancer.

Guan’s research discovered a significant association between mutations in NFE2L2, which encodes the transcription factor Nrf2, and increased resistance to radiotherapy in oral cancer patients undergoing surgery and adjuvant chemo/radiotherapy. Tumors carrying the mutant Nrf2E79Q exhibit elevated expression of chemotactic factors for PMN-MDSCs (such as CXCL1, CXCL3, and CSF3), thereby enhancing the recruitment of PMN-MDSCs and contributing to radiotherapy resistance in oral cancer patients (33). Granulocyte colony-stimulating factor (G-CSF) is an important regulator of neutrophil transport and is notably expressed in various tumors. It plays a key role in the proliferation, migration, and functional maintenance of MDSCs (34). In both *in vitro* and *in vivo* studies, tumor-derived G-CSF has been implicated in the development of chemotherapeutic resistance by promoting an increase in MDSC populations. Consequently, strategies such as splenectomy to deplete MDSC or the administration of anti-Gr-1 antibodies have been shown to sensitize G-CSF-producing tumors to cisplatin (35). The immunosuppressive activity of MDSCs further undermines the efficacy of immunotherapies, by inhibiting T cell and other immune cell functions through various mechanisms. This activity contributes to tumor drug resistance and evasion of immune surveillance (36). MDSCs release immunoregulatory molecules, such as adenosine, TGF-β, and suppressive cytokines, which regulate immune responses and can facilitate the recruitment of Tregs (37). Additionally, MDSCs express the CD39 and CD73, which sequentially convert ATP into adenosine, a critical mediator of immune suppression within the TME (38). Furthermore, MDSCs can enhance the expression of arginase 1 and inducible nitric oxide synthase (iNOS), resulting in the depletion of arginine. This depletion inhibits T cell proliferation and activation, thereby diminishing T cell-mediated anti-tumor activity (39). MDSCs expressing iNOS can modify T cell antigen recognition, impede T cell proliferation, and induce T cell apoptosis through the production of NO, nitrite, and reactive oxygen species (ROS) (40). Additionally, cysteine, a critical amino acid for T cell activation, can be manipulated by MDSCs, which convert extracellular cystine into cysteine without exporting the products, thereby inhibiting T cell activity (41). Immune suppressive factors such as IL-10 and TGF-β are also involved in MDSC-mediated immune suppression. IL-10 has been demonstrated to impair CD8 T cell function across various tumor types (42), while TGF-β undermines T-cell immunity by promoting regulatory T-cell differentiation, further enhancing immunosuppression within the tumor microenvironment (43). The elevated expression of indoleamine 2,3-dioxygenase or arginase 1 in MDSC can also lead to the expansion of Tregs, thereby inhibiting anti-tumor immune responses (44).

### 2.3 Tregs

T cells constitute a vital component of the adaptive immune system, playing a critical role in inhibiting tumor growth through mechanisms such as cytolysis and IFN-γ-mediated cell cycle arrest (45). Within the CD4+ T cell population, there exists a diversity of subsets characterized by pro-inflammatory and anti-inflammatory functions, with Tregs being a major anti-inflammatory subset with potent immunosuppressive capabilities (46). Tregs are essential for ensuring that the immune system generates sufficient inflammatory responses against foreign and novel tumor antigens while concurrently maintaining sufficient anti-inflammatory activity to avert excessive inflammation that could result in tissue damage or mortality. Despite their importance in preserving peripheral tolerance and preventing autoimmunity, Tregs also suppress anti-tumor immunity within the TME (47). Tumor cells or macrophages can recruit Tregs by secreting chemokines (such as CXCL12, CCL17, CCL22, and CCL1) (48). Tregs inhibit immune responses by expressing various cytokines that target tumor cells (49). Compared to patients who are untreated or have undergone surgery alone, those receiving chemoradiotherapy exhibit an increased frequency of CD4+CD39+ Tregs, and the Treg compartment, including cells with enhanced immunosuppressive functions, expands under the capabilities (such as upregulated LAP, GARP, and CD39) (50). In HNSCC, neoadjuvant PD-1/CTLA4 blockade has shown a considerable response rate. Tregs are depleted during chemotherapy with the cytotoxic drug cyclophosphamide. But surviving Tregs proliferate rapidly, thereby inhibiting the development of anti-tumor immunity following lymphocyte depletion (51). TIM-3 is a co-inhibitory molecule that has been found to be upregulated in response to radiotherapy. The cytoplasmic tail of TIM-3 lacks known inhibitory signaling motifs. During radiotherapy and anti-PD-1 (pembrolizumab) combination therapy in HNSCC, TIM-3 was upregulated on Tregs (52). TIM-3-positive Tregs express higher levels of IL-10 than TIM-3-negative Tregs, and they exhibit stronger inhibition of T lymphocytes cell-mediated IFN-γ and TNF-α release. Tregs suppress dendritic cell antigen presentation and induce T cell exhaustion within the tumor microenvironment through the secretion of suppressive cytokines, including TGF-β, IL-35, and IL-10, as well as by modulating the expression of inhibitory receptors. Furthermore, Tregs can directly kill effector T cells or antigen-presenting cells via perforin, granzyme B, or Fas/Fas ligand interactions (53).

The regulation of Treg function and DC activation status is crucial for developing resistance in highly radioresistant tumors. Research has demonstrated that in HNSCC, the population of myeloid cells increases following radiotherapy. In these radioresistant tumors, the combination of radiotherapy with anti-CD25 and anti-CD137 therapies may stimulate the activation of CD103+ DCs within the tumor-draining lymph nodes, leading to a CD8+ T-cell-dependent immune response. Concurrently, Tregs may be reprogrammed into an effector phenotype, enhancing the efficacy of tumor therapy (54). This reprogramming results in a TME that is more inflammatory and less tolerant. Developing novel strategies to augment T cell responses could broaden the scope of anti-cancer therapies. The expression of CD96 has been correlated with improved survival in HPV-positive HNSCC, as its cross-linking activates tumor-infiltrating T cells. Anti-CD96 antibodies exert direct effects on T cells by enhancing gene expression networks associated with T cell activation, leading to T cell proliferation, cytokine secretion, and resistance to Treg-mediated suppression. This highlights the potential of anti-CD96 antibodies in cancer immunotherapy (55).

### 2.4 CAF

CAFs demonstrate significant heterogeneity and can originate from various cell types, including resident fibroblasts, bone marrow-derived mesenchymal stem cells, tumor cells, and endothelial cells (56). Distinct CAF subtypes, characterized by diverse phenotypes and functions, have been identified across different cancers types. These include antigen-presenting CAFs, myofibroblastic CAFs, and inflammatory CAFs (57). CAFs constitute a major component of the TME and perform multiple tumor-promoting functions within this milieu. They play a crucial role in mediating communication among various cells in the tumor stroma (58). The functions of CAFs are extensive and closely related to their environment. Within the TME, CAFs facilitate and promote tumor cell growth, metastasis, and resistance to drug therapy (59). Prolonged stimulation by the TME can irreversibly activate quiescent fibroblasts into CAFs, leading to increased production of ECM and cytokine. In HNSCC, the primary functions of CAFs include modulation of invasion, proliferation, stemness, EMT, and immune response (60).

In HNSCC, CAFs upregulate autophagy by increasing the secretion of IL-6 and IL-8, thereby reducing cellular sensitivity to cisplatin (61). Additionally, CAFs can drive EMT and confer radio resistance to cancer cells. CAF-derived extracellular vesicles, which carry various miRNAs, can influence chemoresistance in HNSCC. For instance, CAFs can secrete elevated levels of miR-876-3p, which inhibits GATA1 expression in OSCC cells, thereby downregulating IGFBP3 and conferring resistance to cisplatin. Notably, IGFBP3 is typically elevated in tumors that are responsive to chemoradiotherapy, while GATA1 is implicated in the regulation of carboplatin resistance and tumorigenesis (62). Moreover, CAFs derived from different patients display variable sensitivities to cisplatin, with recurrent patients’ CAFs requiring significantly higher doses of the drug. Through paracrine signaling, CAFs can either enhance or inhibit the colony-forming capacity and cisplatin resistance of HNSCC cells (63). Additionally, CAFs facilitate the recruitment of Tregs and MDSCs, contributing to immunotherapy resistance. Studies have identified distinct molecular characteristics among various CAF subtypes, suggesting that these subgroups play critical roles in modulating the immunological milieu of human HNSCC. Consequently, these subtypes have the potential to serve as biomarkers for predicting response and resistance in clinical trials (64, 65).

Furthermore, fibroblasts play a pivotal role in the synthesis and deposition of ECM proteins (66). The ECM functions as a structural scaffold essential for maintaining tissue and organ homeostasis and constitutes a crucial component of the cancer microenvironment that facilitates tumorigenesis. The ECM is composed of fibrous and non-fibrous collagens, elastin, proteoglycans, glycoproteins, laminins, and fibronectins (67). Beyond its role in sustaining the ECM and promoting tumor metastasis, alterations in the abundance of ECM components can lead to variations in tissue density and stiffness, potentially influencing resistance to cancer therapies. The excessive production of ECM proteins by CAFs increases the ECM stiffness, primarily toward the tumor core, creating a significant barrier to drug delivery and serving as a predictor of poor prognosis and high recurrence rates (68). For instance, hyaluronic acid (HA), a prominent glycosaminoglycan component of the ECM, interacts with the CD44v^high^ALDH1^high^ subpopulation in HNSCC cells. HA-induced epigenetic modifications, involving histone methyltransferase DOT1L and H3K79 methylation, promote production of miR-10, resulting in an upregulation of RhoGTPase, surviving proteins, CD44v^high^ALDH1^high^ subpopulation, CSC migration/invasion and chemoresistance (69). Therefore, targeting CAFs and the ECM may help overcome tumor resistance in HNSCC.

### 2.5 Additional considerations

Additional elements within the TME contribute to resistance mechanisms in HNSCC and merit further investigation as potential therapeutic targets. Notably, mesenchymal stem cells (MSCs) have been observed to enhance proliferation and motility of HNSCC cells upon interaction. Transplantation of parental head and neck cancer cells, cells fused with MSCs, or cells exposed to MCSs onto the tongue of mice revealed that the development of paclitaxel resistance (70). Furthermore, the EMT process also contributes to resistance within HNSCC. The overexpression of growth factors, AXL, and c-MET in patients with radiation and cisplatin-resistant HNSCC may serve as key drivers of resistance. c-MET, a tyrosine kinase receptor (RTK) activated by hepatocyte growth factor, and AXL, another RTK within the TAM family, facilitate the EMT process. Cabozantinib, an inhibitor targeting VEGF, c-MET, and AXL, exhibits potent inhibitory effects, inducing mitotic catastrophe and apoptosis in radiation and cisplatin-resistant HNSCC cells (71).

## 3 Metabolic substrate-mediated drug resistance in the TME

Cell metabolism encompasses a complex network of biochemical reactions that transform metabolic substrates into essential biological functions, thereby maintaining cellular homeostasis (72). Metabolic demands and preferences undergo significant changes in tumor progression. Metabolic reprogramming in tumor cells is now recognized as a hallmark of cancer (73). In response to genetic mutations and the stressful, ever-changing microenvironment, cancer cells independently reprogram their glucose, amino acid, and lipid metabolism, thereby altering their biological pathways. This metabolic reprogramming enables cancer cells to enhance survival, proliferation, and dissemination, induce angiogenesis, and contribute to tumor resistance (74).

### 3.1 Glycolysis

Glucose metabolism is intricately linked to cancer physiology and pharmacology, serving as a primary source of bioenergy and macromolecules for maintaining cellular balance. In normal human cells within the microenvironment, glucose molecules are metabolized to pyruvate through glycolysis. Pyruvate can undergo further oxidation in the mitochondria through oxidative phosphorylation, producing up to 38 ATP molecules. Under hypoxic conditions, glycolysis predominantly generates organic acids and a reduced amount of 2 ATP. Conversely, tumor cells exploit glycolysis to its fullest extent even in the presence of oxygen, a phenomenon known as the Warburg effect (75). Although glycolysis is less efficient in ATP production compared to oxidative phosphorylation, it proceeds at a faster rate. Malignant cells are characterized by uncontrolled invasive proliferation and inadequate angiogenesis, resulting in increased oxygen consumption, insufficient blood supply, and exacerbated hypoxia within the TME, which subsequently augments glycolysis in tumor cells (76).

Dysregulation of glucose metabolism is a critical factor not only in the process of tumorigenesis but also in treatment resistance and relapse (77, 78). The abnormal activation of glycolysis leads to the accumulation of lactic acid, which drives tumor progression and significantly contributes to tumor acidosis. This acidosis synergistically promotes tumor progression, confers resistance to certain antitumor therapies and impairs antitumor immunity (79, 80). The enhanced Warburg effect improves redox homeostasis, prevents radiation-induced increases in intracellular ROS levels beyond lethal thresholds, induces radio resistance, and enhances DNA repair mechanisms by facilitating nucleotide biosynthesis (81).The Warburg effect also confers resistance to cytotoxic chemotherapeutic agents. Metabolic reprogramming influences numerous signaling pathways associated with resistance to chemotherapy and radiotherapy, including Wnt, PI3K/AKT, Notch, NF-κB, and MAPK, thereby altering the efficacy of combined modality treatments (78). Additionally, oncogene-driven metabolic reprogramming enhances the pentose phosphate pathway and aerobic glycolysis, promoting DNA repair and anti-apoptotic processes. Furthermore, the reprogramming of glucose metabolism plays a significant role in immune resistance in HNSCC. This metabolic reprogramming, in conjunction with hypoxia and acidosis, facilitates oncogene signaling pathways and stromal cell function to maintain energy supply and immune evasion. Elevated lactic acid concentrations inhibit the proliferation and survival of immune cells, such as by disrupting T cell metabolism and antitumor activity, and can increase the proportion of Treg cells, and maintain their immunosuppressive function by up-regulating FOXP3 and MCT1 (82). Lactate influences the functions of dendritic cells and tumor-associated macrophages. Lactic acidosis inhibits NFAT, reduces the production of IFNγ, and downregulates PPARγ, limiting the cell cytolytic function of NK cells and promoting tumor progression. Additionally, lactate derived from tumors can enhance the polarization of macrophages towards the M2 phenotype (83, 84). Lactic acidosis also diminishes the functions of M1 macrophage by downregulating IL-6, iNOS, and CCL2 (85). The acidic TME can further inhibit the secretion of TNF from monocytes, thereby protecting malignant cells from immune clearance (86).

Consequently, targeting the reprogramming of glucose metabolism provides new insights into the treatment of HNSCC. Propranolol, a non-selective β-blocker, exhibits anticancer activity through the inhibition of mitochondrial metabolism (87). However, the response of HNSCC to propranolol involves enhanced glycolysis, which may limit its effectiveness as a monotherapy. The combination of propranolol with the glycolysis inhibitor dichloroacetate (DCA) enhances the effects of chemoradiotherapy and sensitizes resistant cells to cisplatin and radiation (88). Furthermore, pyruvate dehydrogenase kinase-1 (PDK1), a mitochondrial enzyme frequently overexpressed in cancer cells, shifts glucose metabolism from oxidative phosphorylation to aerobic glycolysis (89). In several cetuximab-resistant HNSCC xenograft models, DCA inhibits PDK1 activity within glycolysis. When DCA is administered in conjunction with cetuximab, there is a marked increase in tumor sensitivity to cetuximab, resulting in significant tumor regression, an outcome not achieved with either agent alone (90). OSCC is often characterized by elevated level of EGFR. Erlotinib, a small molecule tyrosine kinase inhibitor, effectively inhibits EGFR activity but frequently encounters resistance. Quercetin, a naturally occurring flavonoid, exhibits anticancer properties across various cancer cell types (91). At a concentration of 5 μM, quercetin effectively inhibits cell growth, reduces glucose utilization, and suppresses cell invasion, thereby resensitizing resistant cell lines to erlotinib (92).

### 3.2 Lipids

Lipids perform essential biological functions in the human body, including energy storage, acting as signaling molecules, and serving as structural components of cellular membranes. Consequently, numerous studies have demonstrated that abnormalities in lipid content, composition, and metabolism are intricately linked to various diseases (93). Lipid metabolic is recognized as a hallmark of tumor metabolism (94). Increasing evidence suggests that lipid metabolism is often enhanced at various stages of cancer progression to satisfy the demands of rapid tumor development. This upregulation can induce alterations in signaling pathways and epigenetic events, as well as facilitate modifications in membrane composition that promote metastasis (95, 96). Lipid metabolism and its products regulate cancer cell growth, survival, proliferation, migration, invasion, and metastasis. Cancer cells also exploit lipid metabolism to modulate cellular activity within the TME to their advantage, thereby enhancing treatment resistance treatment, and promoting recurrence (97).

A characteristic feature of chemotherapy-resistant cancer cell lines is the reduced fluidity of the lipid bilayer in the cell membrane, which impedes drug uptake through passive diffusion or endocytosis (98). Moreover, chemotherapy-resistant cancer cells exhibit a comparatively lower ratio of polyunsaturated fatty acids to saturated fatty acids, rendering them less vulnerable to toxic lipid peroxidation reactions, which can induce apoptosis and ferroptosis (99). This decreased vulnerability to lipid peroxidation adversely affects the efficacy of chemotherapy. Metabolic and expression analyses of radiation-resistant nasopharyngeal carcinoma cells show increased fatty acid oxidation and elevated levels of CPT1A protein compared to radiation-sensitive cells. Inhibition of fatty acid oxidation enhances the sensitivity of resistant cells to radiation (100).

### 3.3 Amino acids

Amino acid metabolism fulfills the growing energy and biosynthetic requirements of tumors. In addition, tumor cells frequently depend on the uptake and/or synthesis of amino acids to support disease progression (101). Several non-essential amino acids limit tumor growth *in vivo (* 102). The dependency of cancer cells on amino acid uptake and metabolism suggests that targeting these processes in specific cell types could serve as a viable cancer treatment strategy, with substantial evidence supporting this notion.

Recent research suggests that reprogramming of amino acid metabolism significantly contributes to tumor resistance mechanisms. Specifically, glutamine metabolism influences the expression of E-cadherin and N-cadherin-critical markers of EMT (103), through the regulation of the MYC transcription factor, thereby enhancing cancer cell resistance to chemotherapy and immunotherapy. The upregulation of the glutamine transporter SLC1A5 in response to radiotherapy results in elevated glutamine levels of in patients with HNSCC. Radiotherapy activates interferon signaling pathways, increasing the expression of interferon regulatory factor 1, which subsequently upregulates transferrin receptors, disrupts intracellular iron homeostasis, and induces ferroptosis in cancer cells, culminating in tumor cell death (104). However, glutamine can inhibit this process, leading to radiotherapy resistance. Additionally, the cystine/glutamate antiporter (xCT) transporter is involved in HNSCC resistance by regulating the import of cystine and export of glutamate (105). xCT is expressed in cancer cells with CD44v expression, which facilitates antioxidant defense through glutathione production, thereby resisting oxidative stress and enhancing resistance to cancer therapies. Sulfasalazine, an inhibitor of xCT-dependent cystine transport, has been shown to effectively reduce tumor growth *in vivo* and eliminate CD44v-expressing undifferentiated HNSCC cells, thereby promoting the efficacy of anti-EGFR treatment on the remaining differentiated cells (106). Furthermore, the sensitivity of HNSCC cells to targeted therapies can be increased by disrupting GSH synthesis and enhancing mitochondrial metabolism, which leads to the generation of ROS and subsequent oxidative damage. Tryptophan undergoes degradation into kynurenine via the catalytic action of indoleamine 2,3-dioxygenase and tryptophan-2,3-dioxygenase, which in turn activates the downstream aryl hydrocarbon receptor (AhR). AhR, a cytoplasmic transcription factor, broadly suppresses function of immune cells, including Tregs, DCs and CD8 T cells (107). Additionally, the amino acid oxidase IL4I1 can suppress T cell responses. Neutralizing IL4I1 activity has the capacity to restore T cell proliferation (108).

Adenosylmethionine (SAM) is a methyl donor with diverse biological roles, demonstrating notable anticancer properties across various malignancies. However, cancer cells often diminish SAM levels through multiple mechanisms within the TME (109). In HNSCC cells, SAM can induce cell cycle arrest, thereby influencing cell motility and invasion of the extracellular matrix (110). Moreover, SAM triggers endoplasmic reticulum stress in HNSCC cells, activates the unfolded protein response, and induces apoptosis (111). It also enhances the sensitivity of HNSCC cells to cisplatin, working synergistically with cisplatin to inhibit cell growth. The combination of anti-CD47 therapy with glutamine blockade during radiotherapy—a strategy in which CD47, an immune checkpoint receptor, shields cells from macrophage phagocytosis—results in significant tumor growth suppression, induction of ferroptosis, and prolonged survival in mouse models (112, 113).

## 4 Current treatment of HNSCC patients

The therapeutic approach for each HNSCC patient is contingent upon factors such as the anatomical site, disease stage and characteristics, functional considerations, and patient preferences (114). Traditional treatment for HNSCC typically involves surgical resection followed by adjuvant radiotherapy or chemoradiotherapy depending on the stage of the disease (115). For patients with small primary tumors without clinical lymph node involvement or with involvement of only a single lymph node, surgery or radiotherapy may be sufficient (116). For tumors with more advanced staging of the primary tumor or lymph nodes, postoperative radiotherapy or chemoradiotherapy guided by pathological risk factors can reduce the risk of recurrence and improve survival rates. The introduction of cisplatin (CDDP) has significantly advanced chemotherapy for HNSCC, with the FP combination therapy (CDDP + 5-fluorouracil [5-FU]) becoming widely adopted (4). Both CDDP and carboplatin, as platinum-based anticancer agents, have been extensively employed since 2000 (117). In case where pathological features such as extracapsular spread, close or positive surgical margins, or perineural invasion suggest an elevated risk, high-dose cisplatin chemotherapy in conjunction with radiotherapy can improve disease-free survival rates (118). Nevertheless, prolonged exposure to cisplatin frequently results in the development of tumor cell resistance, ultimately leading to treatment failure and a poorer prognosis. Given that platinum compounds, particularly cisplatin, constitute the primary first-line chemotherapeutic agents in the clinical management of HNSCC, overcoming cisplatin resistance is crucial for enhancing therapeutic efficacy. Subsequently, two chemotherapy drugs, paclitaxel (PTX) and docetaxel (DTX), which induce cell cycle arrest in cancer cells by preventing microtubule depolymerization, have also been introduced into HNSCC treatment (119). With the frequent use of these two drugs, multiple pathways mediating PTX and DTX resistance have impacted chemotherapy efficacy (120). The EGFR monoclonal antibody cetuximab has been approved by the FDA as a radiosensitizer for the treatment of recurrent or metastatic disease. Although cetuximab has a poorer therapeutic effect on HPV-associated diseases compared to cisplatin, it is commonly used in patients who are not suitable candidates for cisplatin therapy (121). Currently, cisplatin/carboplatin associated with 5-FU and cetuximab, known as the EXTREME regimen, is the first-line treatment for HNSCC patients with locally advanced or recurrent/metastatic (R/M) disease, offering median overall survival of approximately 10 months (122).

Aside from early oral cancers or laryngeal cancers, most HNSCC cases require systemic treatment (123). The emergence of immunotherapy provides a new approach for cancer treatment. Immunomodulatory drugs targeting immune checkpoint pathways play a role in the interactions between tumor cells and T lymphocytes. Immunotherapy is a promising and effective strategy for treating various cancers, including HNSCC (124). In particular, immune checkpoint inhibitors (ICI)) have been applied to HNSCC and significantly improve survival by targeting PD-1, PD-L1, and cytotoxic T lymphocyte-associated protein 4 (CTLA-4) (125, 126). T cells require activation through T cell receptors and co-stimulation through CD28 to become effector T cells that exert immune responses, which is negatively regulated by CTLA-4. PD-L1 expressed on the tumor surface binds to PD-1 on T cells, preventing T cell cytotoxicity, leading to T cell exhaustion or reduced infiltration, and ultimately causing immune escape of the tumor. Therefore, antibodies targeted to CTLA-4 and PD-1 are applied to reactivate T cells and maintain their anti-tumor effect. FDA has approved ICI pembrolizumab and nivolumab for cisplatin-resistant R/M HNSCC. Recent large-scale clinical trials of anti-PD-1/PD-L1 therapies have strengthened the biological rationale for targeting the PD-1/PD-L1 pathway in HNSCC, showing improved results compared to standard care. For R/M HNSCC patients with PD-L1 expression, pembrolizumab with or without chemotherapy can result in a median survival of about 14 months (127). Although immunotherapy, represented by ICIs, is changing the way cancer is treated, there are still significant limitations, such as the fact that only a small percentage of cancer patients can benefit from this treatment, and the fact that cancer cells can develop mechanisms to avoid interacting with immune cells and thus become resistant. There are still a large number of clinical studies being conducted on ICI, and clarifying the mechanisms by which ICI develops resistance provides a direction for improving the efficacy of tumor therapy and finding new and effective ways to treat tumors (**Table 1**).

**Table 1:** Selected clinical trials of immune checkpoint inhibitors in HNSCC in the last 5 years.

Target	Drug	Clinicaltrials.gov Identifier	Phase	Treatment setting
PD-1	Pembrolizumab plus cetuximab	NCT03082534	II	R/M HNSCC
	Pembrolizumab plus cabozantinib	NCT03468218	II	R/M HNSCC
	Pembrolizumab versus cetuximab concurrent with radiotherapy	NCT02707588	II	HNSCC unfit for cisplatin
	Pembrolizumab and Afatinib	NCT03695510	II	R/M HNSCC
	Pembrolizumab with chemoradiotherapy	NCT02586207	IB	Locally advanced HNSCC
	Pembrolizumab plus epacadostat	NCT03358472	III	R/M HNSCC
	Pembrolizumab	NCT02641093	II	Local-regionally advanced HNSCC
	Nivolumab with stereotactic body radiotherapy	NCT02684253	II	Metastatic HNSCC
	Nivolumab	NCT02488759	I/II	Resectable HPV-positive and HPV-negative HNSCC
	Nivolumab	NCT03021993	II	OSCC
	nivolumab alone or combined with ipilimumab	NCT03700905	III	Resectable HNSCC
	Toripalimab combined with gemcitabine and cisplatin	ChiCTR2100043743	Ib	Locally advanced HNSCC
	Camrelizumab and apatinib	NCT04393506	I	Locally advanced resectable OSCC
	Camrelizumab and chemotherapy	ChiCTR1900025303	II	Locally advanced HNSCC
	Budigalimab	NCT03000257	I	HNSCC and NSCLC
	Cemiplimab, radiotherapy, cyclophosphamide, and granulocyte macrophage colony-stimulating factor	NCT02383212	I	R/M HNSCC
PD-L1	SBRT with single-dose durvalumab	NCT03635164	I/Ib	HPV-unrelated locally advanced HNSCC
	durvalumab with or without tremelimumab	NCT02369874	III	R/M HNSCC
	durvalumab plus cetuximab	NCT03691714	II	R/M HNSCC
	durvalumab with cetuximab and radiotherapy	NCT03051906	I/II	Locally advanced HNSCC
	durvalumab plus IRX-2	NCT03381183	Ib	R/M HNSCC
	avelumab, palbociclib, and cetuximab	NCT03498378	I	R/M HNSCC
	avelumab and cetuximab	NCT02938273	I	Advanced HNSCC
CTLA-4	ipilimumab and Nivolumab in combination with radiotherapy	NCT03162731	I	High-risk locally advanced HNSCC

R/M HNSCC, recurrent or metastatic head and neck squamous cell carcinoma; OSCC, oral squamous cell carcinoma; NSCLC, non-small cell lung cancer.

## 5 Future therapeutic strategies to overcome treatment resistance

Many studies are now exploring many new treatment strategies for treatment resistance due to TME (**Table 2**). Innovative therapeutic strategies focusing on TAM are now widely studied in tumor treatment (128). In HNSCC, tumor-recruited and polarized M2 TAMs can secrete C-C motif chemokine ligand 15 (CCL15) via hypoxia-inducible factor (HIF)-2α-dependent pathways. CCL15 then interacts with C-C motif chemokine receptor 1 (CCR1) on tumor cells, activating NF-κB signaling and leading to gefitinib resistance. Investigators found that metformin was found to increase the sensitivity of HNSCC cells to gefitinib both *in vivo* and *in vitro* by inhibiting the expression of CCL15 in hypoxia-enhanced M2-type TAMs, but also on the surface of CCR1 in HNSCC cells (26).In tumors treated with radiotherapy and PD-L1 blockade, immune checkpoint receptor TIM-3 is upregulated on CD8 T cells and Tregs. Combining anti-TIM-3 with anti-PD-L1 and radiotherapy significantly delays tumor growth in HNSCC models, enhances T cell cytotoxicity, reduces Tregs, and improves survival. Targeting Treg depletion restores anti-tumor immunity in mice treated with radiotherapy and dual immune checkpoint blockade, leading to tumor rejection and the induction of immune memory (53). Studies have found that complement system inhibition can play a role in a variety of diseases, including tumors, and have also provided new insights into the treatment of disease (129, 130). Complement system inhibition has been shown to affect HNSCC treatment resistance by impacting Tregs. Inhibition of complement C3a and C5a signaling with receptor antagonists accelerates tumor growth in various HNSCC cell lines and correlates with an increased frequency of Tregs. Therefore, combining targeting of C3a and C5a receptors with anti-Treg therapy might enhance therapeutic advantages (131). When HNSCC cell lines are co-cultured with CAFs, the expression of matrix metalloproteinase-1 is increased in both tumor cells and CAFs, leading to decreased sensitivity of HNSCC to cetuximab. Therefore, the presence of MMP inhibitors can partially eliminate CAF-induced resistance (132). Additionally, studies have shown that CAFs activated by the TGF-β pathway can limit the efficacy of cetuximab *in vitro* and *in vivo*. Blocking the TGF-β pathway with the SMAD3 inhibitor SIS3 can enhance cetuximab efficacy and prevent cetuximab resistance (133). Glucose transporter 1 (GLUT1) facilitates glucose uptake and is overexpressed in most cancers. In HNSCC cells, GLUT1 knockdown reduces glucose uptake, making HNSCC cells more sensitive to cisplatin treatment under both normoxic and hypoxic conditions (134). Among glycolytic enzymes, hexokinase (HK) is the rate-limiting enzyme in the first step of glycolysis, catalyzing glucose to glucose-6-phosphate. HK2, a specific isozyme, is highly expressed in head and neck cancer tissues in mice and humans compared to normal tissues. Inhibition of HK2 in HNSCC cells reduces glycolysis rates and enhances sensitivity to cisplatin and 5-fluorouracil (135). Squalene epoxidase (SQLE) can convert squalene to 2,3-oxidosqualene, acting as an enzyme in the endogenous cholesterol system. It has been identified as a critical driver of chemotherapy resistance and tumorigenesis (136). In HNSCC, Zhao et al. discovered the pivotal role of SQLE in cisplatin resistance. Depletion of SQLE in cisplatin-resistant HNSCC cells significantly suppresses the oncogenic phenotype and enhances sensitivity to cisplatin. Combined treatment with cisplatin and the SQLE inhibitor terbinafine demonstrates strong synergistic effects in patient-derived xenograft models and *in situ* models, significantly increasing drug sensitivity and markedly reducing tumor growth (137). The glutamine transporter ASCT2 is also overexpressed in HNSCC. Knockdown of ASCT2 and combination with small molecule antagonists significantly inhibit intracellular glutamine levels and downstream glutamine metabolism, improving the response of HNSCC to cetuximab (138). Therefore, targeting lipid metabolic reprogramming may offer new strategies for overcoming treatment resistance in HNSCC.

**Table 2:** Some current studies of improving HNSCC treatment outcomes by targeting TME.

Inhibitor	Therapy resistance	Main Target Gene/Protein	Mechanism
Metformin	Gefitinib resistance	CCL15	Inhibiting the expression of CCL15 in hypoxia-enhanced M2-type TAMs
Anti–TIM-3	PD-1/PD-L1 resistance	TIM-3	Enhances T cell cytotoxicity, reduces Tregs
MMP inhibitors	Cetuximab resistance	MMP	Eliminate CAF-induced resistance
SIS3	Cetuximab resistance	SMAD3	Block TGF-beta pathway activates CAF
GLUT1-shRNA	Cisplatin resistance	GLUT1	Reduces glucose uptake
HK2-shRNA	Cisplatin and 5-fluorouracil resistance	HK2	Reduces glycolysis rates
Terbinafine	Cisplatin resistance	SQLE	SQLE inhibition diminishes Akt's binding affinity to lipid rafts, ultimately leading to c-Myc destabilization
V-9302	Cetuximab resistance	ASCT2	Suppress intracellular glutamine levels and downstream glutamine metabolism

## 6 Conclusions

In summary, treatment resistance remains a significant challenge in cancer therapy. This review has synthesized the roles of immune cells, non-immune cells, and metabolic reprogramming within the TME in contributing to treatment resistance in HNSCC. Additionally, it highlights the therapeutic potential of targeting these cellular and metabolic components in HNSCC. The TME of HNSCC is characterized by its heterogeneity and dynamic nature, with diverse cell types and their secreted cytokines forming a complex network. These components of the TME can interfere with various HNSCC treatment modalities, including radiotherapy, chemotherapy, targeted therapy, and immunotherapy, thereby influencing treatment outcomes. Further research is needed to elucidate the mechanisms through which the TME contributes to treatment resistance and to develop novel strategies for targeting and remodeling the microenvironment. In conclusion, substantial evidence highlights the critical role of the TME in modulating HNSCC treatment responses and tumor recurrence. A comprehensive understanding of the TME is crucial for preventing acquired treatment resistance and enhancing cancer therapy.
